# Learning cooking skills at different ages: a cross-sectional study

**DOI:** 10.1186/s12966-016-0446-y

**Published:** 2016-11-14

**Authors:** Fiona Lavelle, Michelle Spence, Lynsey Hollywood, Laura McGowan, Dawn Surgenor, Amanda McCloat, Elaine Mooney, Martin Caraher, Monique Raats, Moira Dean

**Affiliations:** 1 Institute for Global Food Security, School of Biological Sciences, Queen’s University Belfast, Belfast, BT9 5AG UK; 2 Department of Hospitality and Tourism Management, Ulster Business School, Ulster University, Coleraine, UK; 3 Department of Home Economics, St. Angela’s College, Sligo, Ireland; 4 Centre for Food Policy, Department of Sociology, School of Arts and Social Sciences, City University London, London, UK; 5 Food, Consumer Behaviour and Health Research Centre, School of Psychology, University of Surrey, Surrey, UK

**Keywords:** Learning, Cooking skills, Child, Teenager, Adult, Source, Diet quality, Disease prevention

## Abstract

**Background:**

Cooking skills are increasingly included in strategies to prevent and reduce chronic diet-related diseases and obesity. While cooking interventions target all age groups (Child, Teen and Adult), the optimal age for learning these skills on: 1) skills retention, 2) cooking practices, 3) cooking attitudes, 4) diet quality and 5) health is unknown. Similarly, although the source of learning cooking skills has been previously studied, the differences in learning from these different sources has not been considered. This research investigated the associations of the age and source of learning with the aforementioned five factors.

**Methods:**

A nationally representative (Northern/Republic of Ireland) cross-sectional survey was undertaken with 1049 adults aged between 20–60 years. The survey included both measures developed and tested by the researchers as well as validated measures of cooking (e.g. chopping) and food skills (e.g. budgeting), cooking practices (e.g. food safety), cooking attitudes, diet quality and health. Respondents also stated when they learnt the majority of their skills and their sources of learning. The data was analysed using ANOVAs with post-hoc analysis and Chi^2^ crosstabs with a significance level of 0.05.

**Results:**

Results showed that child (<12 years) and/or teen (13–18 years) learners had significantly greater numbers of, and confidence in, their cooking and food skills, cooking practices, cooking attitudes, diet quality (with the exception of fibre intake where adult learners were higher) and health. Mother was the primary source of learning and those who learnt only from this source had significantly better outcomes on 12 of the 23 measures.

**Conclusions:**

This research highlights the importance of learning cooking skills at an early age for skill retention, confidence, cooking practices, cooking attitude and diet quality. Mother remained the primary source of learning, however, as there is a reported deskilling of domestic cooks, mothers may no longer have the ability to teach cooking skills to the next generation. A focus on alternative sources including practical cooking skills education starting at an early age is required. This study also highlights the need for further longitudinal research on the impact of age and source of learning on cooking skills.

## Background

### Importance of cooking skills

Cooking is a valuable life skill which is often linked with improved diet quality, such as improving the uptake of fruit and vegetables and an increased recognition of healthier foods [1, 2]. In a UK survey of 2000 residents, ‘*learn to cook’* was rated as the fifth most important life skill for modern living (the highest non-tech skill) following ‘*searching the internet,*’ ‘*operating a mobile phone*,’ ‘*connecting WiFi’* and ‘*mastering online banking,’* [3], demonstrating public interest in learning cooking skills.

Cooking skills (CS) have been increasingly used on their own and as part of other initiatives as a preventive measure to address diet-related diseases including obesity [4, 5]. In recent years there has been an increase in CS and food skills (FS) interventions as a means to improve dietary outcomes [2]. Current reviews on cooking intervention studies indicate that these interventions vary in their teaching methods such as information provision, demonstrations and practical hands on sessions and have been targeted at people of all ages, including children [6], teenagers [7], and adults [8]. While the rationale for targeting each of these groups is clearly stated in each intervention, the optimal age for learning CS with regards to cooking skill maintenance and dietary outcomes is yet unknown.

### Rationale for cooking skills interventions with different age groups

The increase in cooking interventions for children may be attributed to the belief that prevention of chronic diseases should begin at an early age [9]. Dave and colleagues [10], noted that a dislike for cooking is associated with fast food consumption which in turn has been linked with increasing levels of obesity. Thus there is an argument for children to learn cooking skills in their developmental years [11]. Among adolescents, those involved in food preparation have been shown to have a higher diet quality than those with no involvement in their meal preparation [12]. In the adult population increased and/or improved cooking skills have been shown to improve cooking attitudes, confidence, healthy food choices and dietary outcomes [2, 8].

Although there has been some success in improving certain aspects of diet and food behaviours in each of these groups in the short term [6, 8, 12], there have been few long term positive changes [2]. Limited evidence shows that cooking behaviours track from adolescence to young adulthood [13], however, it is unknown whether these behaviours can track from childhood right through to adulthood. Thus very little is known about the optimal age for learning CS and its subsequent impact on adult dietary habits.

### Impact of age on the acquisition of skills in other areas

In relation to general skills acquisition, Janacsek *et al*. [14] showed that the most effective time for learning new skills is from childhood until early adolescence. This principle has been shown to be effective in other areas such as education, where early acquisition of learning-related skills has had a positive impact on academic trajectories in math and reading [15]. In light of this background in other life skills, there is a need to investigate whether early learning of CS has a positive dietary impact in later life.

### Measures used in cooking skills research to measure impact

The learning and use of CS and FS have been positively linked to cooking confidence [16], cooking ability [16], food safety [17], reduction in food waste [18] and time spent in meal preparation [19]. The possession of CS has also been associated with cooking identity [20], Creativity [21], and Food Neophilia [22]. As previously emphasised, CS have been associated with better diet quality [23], where diet quality was measured using different instruments such as Eating Choice Index (ECI) [24] and food frequency measures such as Dietary Instrument for Nutrition Education (DINE) [25]. Further, the impact of learning cooking skills and home meal preparation behaviours on BMI has been previously investigated [26]. Utilising these findings, this research investigates the relationships between the age at which the majority of CS are learnt and: (1) current cooking and food skills; (2) current cooking practices; (3) cooking attitudes; (4) diet quality; and (5) health and wellbeing.

### Does source of learning have an impact?

In addition to the timing of learning CS, where or from whom individuals learn their CS also has the potential to influence their dietary habits [27]. Previous research has cited ‘the Mother’ as the primary source for learning CS across all social classes, with cooking classes in school being the second most common source for learning [27]. However, the impact the source of learning has on current cooking and dietary habits is unknown. Thus, a secondary aim of this study was to investigate whether the source of learning also affects the aforementioned five variables.

## Methods

### Procedure and sample

The data reported here were part of a larger cross-sectional survey investigating CS and FS, sociodemographic and psychological factors on diet quality on the island of Ireland (IOI includes Northern Ireland [NI] and Republic of Ireland [ROI]). A nationwide market research company, SMR, conducted all sampling and field data collection. A sample of 1049 adults between the ages of 20–60 years, was selected using quota sampling to ensure the sample was nationally representative. Quotas were applied for gender, age, area of residence and socio-economic grouping to achieve a balance of participants. Participants were eligible if they prepared a main meal at least once a week and only one participant per household was eligible to partake in the study. All survey data were collected using Computer Assisted Personal Interviewing (CAPI) and conducted by fully briefed interviewers in the participants’ home between October and December 2014. Where a potential eligible participant declined to partake, the reason was recorded. A total of 123 potential participants did not partake due to: dietary restrictions which impacted their food choices; being too busy; not being interested; not having a sufficient level of English; without reason; not being aged 20–60 years; never preparing or cooking a main meal; security reasons; and not living at the address (in weighted descending order). All participants were informed that by partaking in the survey they were consenting for their data to be used. No personal details, for example name, were recorded, and participants were made aware that that they could withdraw at any time. Ethical approval for this research was received from Queen’s University Belfast Research Ethics Committee and the study was conducted in line with the guidance given in the Declaration of Helsinki.

### Survey and measures

The survey included a number of development stages; a rapid review of the literature [2], interviews with experts who worked on healthy eating and cooking and food skills education (*n* = 4), and extensive piloting with a range of participants including students, employed, and unemployed adults (*n* = 40), resulting in a number of amendments. Where possible existing reliable and validated instruments were used for some components of the survey, otherwise researcher developed measures were used. An overview of the measures used is given below. Socio-demographic information such as age, gender, education level and occupation of highest household earner was collected (Table 1).

**Table 1:** Characteristics of 3 groups in stages of most learning

	Child			Teen			Adult		
	*N* = 198 (22.9 %)			*N* = 286 (33.1 %)			*N* = 381 (44 %)		
	*Mean*	*SD*		*Mean*		*SD*	*Mean*		*SD*
Age (yrs)	43	12		39		12	41		11
BMI	24.06	4.66		24.62		3.95	24.80		3.69
Household Size	3.17	1.42		3.06		1.30	3.08		1.33
	*N (%)*	*N (%)*		*N (%)*		*N (%)*	*N (%)*		*N (%)*
Sex	Male 37 (18.7)	Female 161 (81.3)		Male 97 (33.9)		Female 189 (66.1)	Male 203 (53.3)		Female 178 (46.7)
SES*	High 91 (46)	Low 107 (54)		High 142 (49.7)		Low 144 (50.3)	High 197 (51.7)		Low 184 (48.3)
	*N (%)*	*N (%)*	*N (%)*	*N (%)*	*N (%)*	*N (%)*	*N (%)*	*N (%)*	*N (%)*
Education Level	Compulsory 33 (16.7)	Further 117 (59.1)	Higher 48 (24.2)	Compulsory 29 (10.1)	Further 184 (64.3)	Higher 73 (25.5)	Compulsory 48 (12.6)	Further 239 (62.7)	Higher 94 (24.7)

*Socio-economic groupings were created based upon the occupation of the highest earner in the household and were classified as High (ABC1 - higher, intermediate, supervisory, clerical & junior managerial, administrative, professional occupations) versus low (C2DE - skilled, semi-skilled and unskilled manual occupations, unemployed and lowest grade occupations), developed by the ONS, UK Office for National Statistics [53]

### Learning of cooking skills

Participants were asked ‘*At what stage of your life did you learn most of your cooking skills*?’ The responses were classified into ‘As a child (under 12 years),’ As a teenager (13–18 years),’ or ‘As an adult (18+ years).’ No example of what was considered ‘most’ was given, because if specific skills or a certain number of skills were suggested, a participant may have responded with the time they learnt a specific skill not when the majority of their learning occurred. In addition, if a certain number of skills was given as an example, this may have excluded participants who had not yet reached that number of skills. By leaving this question as subjective, it allowed for the maximum number of participants to partake and for each participant to respond with a specific time which they considered had the most significant amount of learning. Participants were also asked about from whom and/or where they learnt these skills (multiple sources were allowed).

### Cooking and food skills

Two scales were developed and piloted as part of the survey; the CS scale and the FS scale. These scales were developed so that the measures for CS and FS were culturally appropriate for the IOI. Participants were shown cards with a list of CS, such as chopping, mixing and stirring foods, stewing food, roasting food, and a list of FS for example, preparing meals in advance and comparing prices before buying food. Participants selected those skills which they possessed and were subsequently asked to rate their confidence in these selected skills, using a scale of 1 (very poor) to 7 (very good). The two scales had an acceptable internal reliability (Cronbach’s alpha for both was > .90). These questions resulted in four measures; (1) number of CS; (2) number of FS; (3) CS confidence and; (4) FS Confidence. The number of skills measures were a sum of the number of reported skills and the confidence scores consisted of a mean score. A higher score in each means a higher number of skills used or a higher level of confidence. For further details on scales please contact authors.

### Cooking practices

The food safety measure consisted of a number of questions relating to best practise for the safe handling and storage of food, for example, *“Leftovers should be stored in the fridge and used within: 1 day, 3 days, 5 days, don’t know”* and *“Raw chicken should always be washed before cooking: True, False, Don’t know,”* with a higher score (number of correct answers) equating to a better knowledge and practise in food safety applications. This measure was developed and tested by the researchers (Cronbach’s alpha = 0.62) as there were no short measures available for food safety in food preparation, suitable for the survey. The food waste measure was based on two questions, *“How often do you:* (1) *Throw away food after meals because you make too much*, and (2) *Throw away food because it goes past its use-by or best before date,”* respondents were able to answer either ‘Never,’ ‘Sometimes,’ or ‘Often’, a lower score in food waste equating to being less wasteful with food. This measure had an acceptable internal reliability (Cronbach’s alpha = 0.71). Participants were also asked *“How long do you typically spend preparing and cooking food from start to finish for the main meal on a week day and on a weekend (in minutes)”* to capture difference between midweek and weekend meal preparation patterns.

### Cooking attitudes

Cooking identity i.e. the degree to which you see yourself as a good cook, and Food Neophilia i.e. an openness to trying new foods, were assessed using an adjusted 11 item scale from previous research [20, 28, 29]. A higher score on both of the scales indicates a positive result, i.e. that the participant identified themselves as a good cook, or had a higher willingness to try new foods or techniques. The creativity measure was a composite score (Cronbach’s alpha = 0.78) and related to imagination/creativity with food and cooking. It was measured using 6 statements on a 5-point Likert scale from 1 strongly agree to 5 strongly disagree. A higher score in the measure equated to being more creative.

### Diet quality

Respondent’s likelihood of choosing healthy food options was measured using a validated tool; the ECI [24]. The previously validated DINE was used to assess dietary intake of fibre; saturated fat; fruit and vegetables; fried food; and biscuits, chocolate, or savoury snacks [25]. The frequency of consumption of fried food and biscuits, chocolate, or savoury snacks were converted into a score with a lower score equating to never or very little consumption of the food and a higher score equating to a more frequent consumption. Participants were also asked *“In a typical week how often do you….* (1) *eat take-away foods or fast food which are ready to eat as your main meal (e.g. Chinese, fish and chips or McDonalds etc.) [Consumption of take-away food]* and (2) *eat take-away foods or fast food bought from the supermarket to be eaten at home as your main meal (e.g. Indian meal kits or pizza) [Consumption of convenience food]?”* This provided an indication of how often they did not cook. A higher score in these questions equated to a more frequent consumption of these foods. Participants reported the most common main meal that they prepared and following this were asked to choose the option that best described how they prepared this dish from six options denoting different levels of preparation and cooking options; 1) Buy it ready-made and reheat it; 2) Use mostly pre-prepared ingredients and I assemble the dish; 3) Use mostly pre-prepared ingredients and some fresh, basic or raw ingredients; 4) Use mostly fresh, basic or raw ingredients and some pre-prepared ingredients; 5) Use only fresh, basic or raw ingredients; 6) I do something else not listed here. Responses were classified into 3 categories: mainly fresh ingredients; a mixture of fresh and pre-prepared ingredients; and mainly pre-prepared ingredients.

### Health and wellbeing indicators

BMI was calculated from self-reported data on height and weight. The existing measure was used for health consciousness (General Health Interest [GHI]) [30]. Items from this measure relating to food were used in the survey. Participants responded to two statements, *“I am very particular about the healthiness of food I eat,”* and *“I eat what I like and I do not worry much about the healthiness of food”* on a 5 point Likert scale. A higher score in this measure equated with a greater health motivation.

### Data analysis

All data were analysed using IBM SPSS Statistics Version 22 (IBM Corporation, 2013). Descriptive statistics (means, standard deviations (SD), etc.) were conducted to examine socio-demographic differences between the three groups, Child Learners (CL) [<12 years], Teenage Learners (TL) [12–18 years] and Adult Learners (AL) [18 + years]. Chi2 crosstabs and ANOVAs with post hoc comparisons made with Tukey’s HSD (honestly significant difference) test were used to investigate significant differences between 1) the three groups of learners and 2) the different sources of learning on the different components of CS and FS; Cooking Practices; Cooking Attitudes; Diet Quality; and Health and Wellbeing indicators. Differences were considered as significant for all analysis, at a level of 0.05.

## Results

The sociodemographic details of the three groups (CL, TL, AL) are displayed in Table 1. As seen in Table 1 the mean of all sociodemographic details are similar, with the exception of gender, as the percentage of males increases from CL to TL and then again from TL to AL (18.7 %, 33.9 %, 53.3 % respectively).

Table 2 shows an overview of the significant differences for the ANOVAs performed. Following is a more detailed report of the findings for each component.

**Table 2:** Differences in mean scores between Child, Teen and Adult learners on various measures

	Reliability	Range	Overall Sample	F (df)	Significance	Child (*n* = 198)	Teen (*n* = 286)	Adult (*n* = 381)
	α		M (SD)		P	M (SD)	M (SD)	M (SD)
Cooking + Food Skills								
Cooking Confidence	0.93	0–98	47.78 (29.32)	9.82 (2,862)	0.000	53.40^b^ (27.30)	57.37^a^ (31.64)	47.76^b^ (25.44)
No. of CS	–	0–14	8.21 (4.49)	5.47 (2,862)	0.004	9.31^a^ (3.98)	9.36^a ^(4.55)	8.39^b^ (4.05)
FS Confidence	0.94	0–133	45.82 (38.64)	16.47 (2,862)	0.000	46.50^b ^(35.97)	61.26^a ^(46.13)	44.81^b^ (32.89)
No. of FS	–	0–19	7.83(6.01)	11.64 (2,862)	0.000	7.86^b^ (5.34)	9.96^a^ (6.75)	7.91^b^ (5.52)
Cooking Practices								
Time spent cooking midweek	–	0–240	45.48 (34.02)	21.50 (2,853)	0.000	61.99^a^ (45.73)	46.38^b^ (27.44)	42.87^b ^(30.188)
Time spent cooking weekend	–	0–280	53.83 (36.52)	10.75 (2,862)	0.000	67.62^a^ (44.56)	54.20^b^ (31.28)	53.61^b^ (35.91)
Food Safety	0.62	0–5	2.78 (1.54)	10.98 (2,862)	0.000	3.07^a^ (1.40)	3.15^a^ (1.50)	2.64^b^ (1.52)
Food Waste	0.71	2–6	3.42 (1.00)	7.03 (2,862)	0.001	3.21^a^ (0.96)	3.54^b^ (0.82)	3.46^b^ (1.06)
Cooking Attitudes								
Cooking Creativity	0.78	6–30	18.69 (4.79)	35.28 (2,862)	0.000	20.66^a^ (4.30)	20.21^a^ (4.21)	17.90^b^ (4.52)
Cooking Identity	0.88	7–35	24.45 (5.39)	18.43 (2,862)	0.000	26.19^a^ (4.73)	26.12^a^ (4.47)	24.20^b^ (4.84)
Food Neophilia	0.74	3–15	10.47 (2.67)	13.51 (2,861)	0.000	10.60^b^ (2.70)	11.40^a^ (2.35)	10.42^b^ (2.42)
Diet Quality								
ECI	–	4–20	12.22 (2.95)	3.60 (2,862)	0.028	12.91^a^ (2.99)	12.21^b^ (2.71)	12.50^ab^ (2.89)
DINE (Sat Fat)	–	8–92	35.54 (13.04)	0.87 (2,862)	0.432	35.99 (14.01)	34.46 (10.95)	35.03 (13.00)
DINE (fibre)	–	6–91	34.62 (11.25)	10.73 (2,862)	0.000	35.19^ab^ (9.68)	33.41^b^ (10.61)	37.35^a^ (11.07)
Fried food	–	1–5	2.43 (0.78)	6.84 (2,862)	0.001	2.35^ab^ (0.80)	2.29^a^ (0.70)	2.49^b^ (0.74)
Biscuits/Chocolate/Savoury Snacks	–	1–5	3.13 (1.07)	7.78 (2,862)	0.000	3.14^b^ (1.03)	2.91^a^ (1.04)	3.24^b^ (1.10)
Consumption of Takeaway	–	1–6	2.55 (0.92)	4.75 (2,862)	0.009	2.27^a^ (0.87)	2.49^b^ (0.87)	2.48^b^ (0.87)
Consumption of Takeaway style food	–	1–6	2.29 (0.97)	2.92 (2,862)	0.051	2.07^a^ (0.88)	2.20^ab ^(0.89)	2.26^b^ (0.95)
Portions of Fruit per day	–	0–5	2.48 (0.97)	3.71 (2,838)	0.034	2.69^a ^(1.04)	2.59^ab^ (0.87)	2.47^b^ (0.94)
Portions of Veg per day	–	0–5	1.88 (1.00)	0.60 (2,862)	0.547	1.98 (1.02)	2.01 (0.97)	1.93 (1.00)
Health + Wellbeing Indicators								
BMI	–	13.03–46.42	24.45 (4.20)	1.62 (2,617)	0.254	24.06 (4.66)	24.62 (3.95)	24.80 (3.69)
GHI	–	2–10	6.85 (1.62)	3.92 (2,862)	0.020	7.27^a^ (1.52)	6.90^b^ (1.46)	6.97^ab^ (1.52)

*M* mean, *SD* Standard Deviation, *CS* Cooking skills, *FS* Food skills, *ECI* Eating Choice Index, *DINE* Dietary Index for Nutrition Education, *Sat* Saturated, *BMI* Body Mass Index, *GHI* General Health Interest

Superscript letters depict where significant differences (*P* < 0.05) fall between the groups

### Cooking and food skills

This component consisted of CS and FS confidence and number of CS and FS used by the participants. TL had a significantly higher CS Confidence (F = 9.82 (2,862), *P* < 0.005), FS Confidence (F = 16.47 (2,862), *P* < 0.005) and number of FS (F = 11.64 (2,862), *P* < 0.005), than CL or AL. The number of CS was significantly higher (F = 5.47 (2,862), *P* < 0.005) for both TL and CL in comparison to AL.

### Cooking practices

CL and TL scored significantly higher than AL on the food safety score (F = 10.98 (2,862), *P* < 0.005). CL wasted significantly less food (F = 7.03 (2,862), *P* < 0.005) than TL or AL. Further, CL invested a significantly greater amount of time cooking [both weekday (F = 21.50 (2,853), P < 0.005) and weekend (F = 10.75 (2,862), *P* < 0.005)] than both TL and AL.

### Cooking attitudes

Significant differences were seen between the three groups on all Cooking Attitudes. CL and TL scored significantly higher than AL on Creativity (F = 35.28 (2,862), P < 0.005) and cooking identity (F = 18.43 (2,862), *P* < 0.005). However, TL were more open to new foods (F = 13.51 (2,861), *P* < 0.005) than CL or AL.

### Diet quality

In the ECI, CL had significantly higher scores (F = 3.60 (2,862), *P* < 0.05), indicating a greater interest in eating healthily compared to TL. No differences were seen across the three groups for Saturated Fat intake, although, AL had a significantly higher intake of fibre (F = 10.73 (2,862), *P* < 0.005) than TL. Significant differences were found between the groups on the frequency of consumption of fried food (F = 6.84 (2,862), *P* < 0.005), and on consumption of biscuits, chocolate or savoury snacks (F = 7.78 (2,862), *P* < 0.005). In relation to fried food, TL consumed it less frequently than AL. TL also consumed biscuits, chocolate or savoury snacks less frequently than either CL or AL. A significant difference (F = 4.75 (2,862), P < 0.05) was found between the three groups on the frequency of consumption of take-away, with CL consuming takeaway less frequently than both TL and AL. A bordering significant difference (F = 2.92 (2,862), *P* = 0.05) was found between the groups on the frequency of consumption of take-away styled foods purchased from a supermarket such as an Indian meal kit or pizza (convenience products). Again, CL consumed convenience food less frequently than AL.

Differences between groups on daily fruit and vegetable intakes showed a significant difference on the portions of fruit consumed per day (F = 3.71 (2,838), *P* < 0.05), with CL consuming a significantly higher amount of fruit than AL. No group differences were found on the portions of vegetables consumed (F = 0.60 (2,862), *P* = 0.55).

**Fig. 1:**
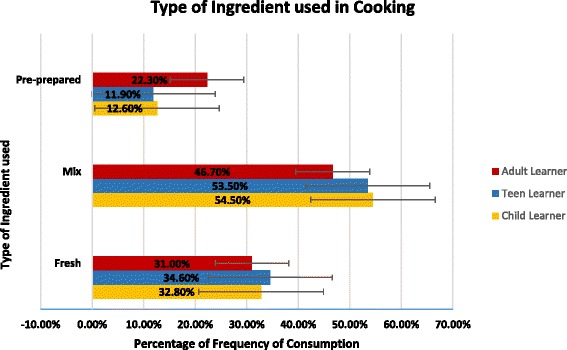
Differences in the type of ingredients used in cooking between Child, Teen and Adult Learners

Figure 1 shows the significant differences (*P* < 0.05) found between the three groups on the type of ingredients used in meal preparation (mainly fresh ingredients, a mixture of fresh and pre-prepared ingredients, mainly pre-prepared ingredients). AL appear to be twice as likely to use pre-prepared ingredients in meal preparation when compared to those who learnt cooking skills at earlier stages who were more inclined to use a mixture of fresh and prepared or fresh ingredients.

### Health and wellbeing indicators

There were no significant differences in BMI across the three types of learners (F = 1.62 (2,617), *P* < 0.2). In relation to GHI, CL had significantly more interest in their health (F = 3.92 (2,862), *P* < 0.05), compared to TL.

### Sources of learning cooking skills

Following is an overview of sources of learning CS and whether they had associations with the same measures as above in Cooking and Food Skills; Cooking Practices; Cooking Attitudes; Diet Quality; and Health and Wellbeing. Table 3 shows the sources from where cooking skills were learnt. The top five stated sources were: Mother (60.1 %); a different relative (16.2 %); Friends (13.6 %); Secondary School (9.3 %); and Food Packet (7.1 %).

**Table 3:** Sources of learning cooking skills

Source	Percent
Mother	60.1
Home (from a different relative)	16.2
Friends	13.6
Post – Primary school (Home Ec etc.)	9.3
Food Packet	7.1
Partner	5.6
Self-taught	5.5
Cookery class	4.7
Internet Websites	4.0
Recipe books/magazines	2.6
TV Programmes	1.3
Don’t know	1.3
Other	1.0
Doctor	0.9
Health promotion leaflet	0.6
Health Practitioner (Dietician)	0.5
Smart Phone App	0.5
YouTube	0.4
Primary School	0.3
Social Media	0.2
Work	0.2
Weight Loss programme	0.1

**Fig. 2:**
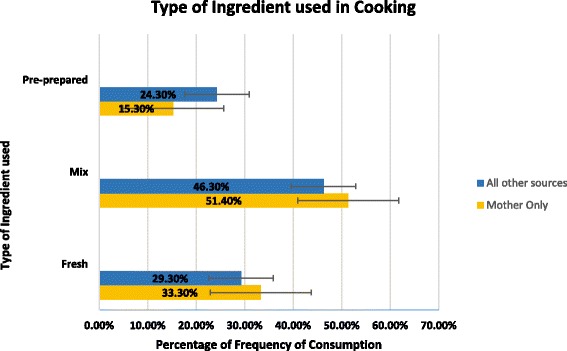
Differences in the type of ingredients used in cooking between Mother and other sources

As participants were able to report more than one source and the overwhelming majority reporting ‘Mother’ as their source of learning, the results are categorised as ‘Mother Only’ (Mother mentioned exclusively) (*N* = 426, 40.6 %) versus any other source or combination of sources of learning (*N* = 600, 57.2 %). Table 4 shows an overview of the significant differences between the sources of learning on current cooking and food skills, Cooking Practices, Cooking Attitudes, Diet Quality and Health and Wellbeing. Results show that Mother only had significantly better outcomes on twelve of the twenty three measures; Cooking Confidence (F = 5.35 (1,1024), *P* < 0.05), Number of CS (F = 6.88 (1,1024), *P* < 0.05), Cooking Creativity (F = 9.03 (1,1024), *P* < 0.005), Cooking Identity (F = 14.40 (1,1024), *P* < 0.005), ECI (F = 5.87 (1,1024), *P* < 0.05), Consumption of fried food (F = 10.58 (1,1024), *P* < 0.005), Consumption of takeaway (F = 13.19 (1,1024), *P* < 0.005), Consumption of takeaway style food from shops (F = 13.46 (1,1024), *P* < 0.005), portions of fruit per day (F = 7.48 (1,998), *P* < 0.05), and portions of veg per day (F = 14.52 (1,1024), *P* < 0.005). There was one borderline significant difference (F = 3.67 (1,1024), *P* = 0.052) for DINE fibre. A significant difference was found also (*P* < 0.005) between source of learning and type of ingredients used in cooking (Fig. 2).

**Table 4:** Differences in mean scores between the Mother only learners and other sources on various measures

	Reliability	Range	Overall Sample	F (df)	Significance	Mother Only (*n* = 426)	Other (*n* = 600)
	α		M (SD)		P	M (SD)	M (SD)
Cooking + Food Skills							
Cooking Confidence	0.93	0–98	47.78 (29.32)	5.35 (1,1024)	0.021	51.31^a^ (27.66)	47.11^b^ (29.39)
No. of CS	–	0–14	8.21 (4.49)	6.88 (1,1024)	0.009	8.85^a^ (4.29)	8.12^b^ (4.40)
FS Confidence	0.94	0–133	45.82 (38.64)	0.29 (1,1024)	0.581	47.52 (35.75)	46.20 (40.44)
No. of FS	–	0–19	7.83(6.01)	0.89 (1,1024)	0.339	8.19 (5.73)	7.83 (6.15)
Cooking Practices							
Time spent cooking midweek	–	0–240	45.48 (34.02)	3.07 (1,1014)	0.092	48.26 (38.45)	44.46 (30.61)
Time spent cooking weekend	–	0–280	53.83 (36.52)	1.95 (1,1024)	0.163	56.42 (39.36)	53.20 (34.25)
Food Safety	0.62	0–5	2.78 (1.54)	1.23 (1,1024)	0.268	2.88 (1.48)	2.77 (1.55)
Food Waste	0.71	2–6	3.42 (1.00)	3.23 (1,1024)	0.072	3.35 (0.97)	3.46 (1.01)
Cooking Attitudes							
Cooking Creativity	0.78	6–30	18.69 (4.79)	9.03 (1,1024)	0.002	19.34^a^ (4.58)	18.44^b^ (4.84)
Cooking Identity	0.88	7–35	24.45 (5.39)	14.40 (1,1024)	0.000	25.38^a^ (4.61)	24.13^b^ (5.55)
Food Neophilia	0.74	3–15	10.47 (2.67)	0.16 (1,1023)	0.686	10.48 (2.57)	10.55 (2.71)
Diet Quality							
ECI	–	4–20	12.22 (2.95)	5.87 (1,1024)	0.016	12.55^a^ (2.93)	12.10^b^ (2.90)
DINE (Sat Fat)	–	8–92	35.54 (13.04)	0.00 (1,1024)	0.995	35.53 (12.97)	35.52 (13.06)
DINE (fibre)	–	6–91	34.62 (11.25)	3.67 (1,1024)	0.052	35.65^a^ (10.54)	34.29^b^ (11.60)
Fried food	–	1–5	2.43 (0.78)	10.58 (1,1024)	0.001	2.33^a ^(0.78)	2.49^b^ (0.76)
Biscuits/Choc/Savoury Snacks	–	1–5	3.13 (1.07)	1.33 (1,1024)	0.249	3.18 (1.09)	3.10 (1.06)
Consumption of Takeaway	–	1–6	2.55 (0.92)	13.19 (1,1024)	0.000	2.40^a^ (0.88)	2.61^b^ (0.92)
Consumption of Takeaway style food	–	1–6	2.29 (0.97)	13.46 (1,1024)	0.000	2.14^a^ (0.91)	2.36^b^ (0.97)
Portions of Fruit per day	–	0–5	2.48 (0.97)	7.48 (1,998)	0.006	2.60^a^ (0.95)	2.43^b^ (0.97)
Portions of Veg per day	–	0–5	1.88 (1.00)	14.52 (1,1024)	0.000	2.04^a^ (1.05)	1.80^b^ (0.95)
Health + Wellbeing Indicators							
BMI	–	13.03–46.42	24.45 (4.20)	0.40 (1,742)	0.528	25.54 (4.39)	24.34 (4.10)
GHI	–	2–10	6.85 (1.62)	3.30 (1,1024)	0.069	6.99 (1.56)	6.81 (1.63)

*M* mean, *SD* Standard Deviation, *CS* Cooking skills, *FS* Food skills, *ECI* Eating Choice Index, *DINE* Dietary Index for Nutrition Education, *Sat* Saturated, *BMI* Body Mass Index, *GHI* General Health Interest

Superscript letters depict where significant differences (*P* < 0.05) fall between the Mother only learners and all other sources

## Discussion

To our knowledge, this is the first study to investigate the associations between *when* CS are learnt (self-perceived measurement of when the majority of learning occurred) and current adult dietary and cooking behaviours. The results indicate that, learning CS mainly at a younger age has a positive effect on many cooking related behaviours, practices and dietary quality.

CL and TL reported better outcomes than AL on the number of currently used CS, cooking creativity and cooking identity. Both CLs and TLs possessed a significantly greater number of CS than AL. Laska et al. [13] showed CS track from adolescence into adulthood. Our study suggests this tracking could start from childhood into adulthood. The possession and application of a greater number of CS has also been linked to a higher diet quality in past studies [2, 23]. In addition, our findings suggest if CS are learnt at younger ages these individuals are more creative in the kitchen and are more likely to see themselves as ‘cooks’ (cooking identity).

CLs spent more time than ALs in food preparation (both weekday and weekend), reported less food waste, consumed less takeaways and takeaway convenience food from the supermarket and shops, and ate more fruit. This suggests that those who learned cooking skills as a child spend more time cooking and are less reliant on convenience food or takeaway. Ultra-processed convenience foods have been shown to be typically high in sugars, fats and sodium [33–36]. CLs and TLs used less of these ingredients in their meal preparation. The consumption of take-away and ready meals are shown to be associated with being overweight and being obese [11, 31]. Thus the learning of CS at an earlier age could be argued as a possible way of combating overweight and obesity, however, further research is needed to investigate this, as our findings, found no difference between the groups on the self-reported BMI measure.

CL also spent more time in meal preparation which would suggest that they are cooking from basic ingredients or making more elaborate meals. Alternatively, this could mean that they are less confident in the kitchen and therefore spend more time in the assembly of convenience products. However, as learning CS at a younger age has been linked with higher confidence scores and generally a better diet quality, we would argue that the reason for more time in the kitchen is more likely associated with cooking from basic ingredients, than a lack of confidence. Other research has found that a greater exposure to fresh ingredients and cooking at a younger age increases the likelihood that this type of food will be consumed through the life course [37, 38]. In addition, in this study CL had an increased consumption of fruit, which may also play a role in weight maintenance [39] and has an inverse association with mortality [40]. Overall, the results suggest learning CS at a younger age may have a role in weight maintenance over the life course, however, as these results are correlations, further studies are needed to investigate the causal relationships.

CLs had significantly better scores in food management and food waste reduction than TL and AL. This shows that the earlier CS are learnt, the less food is wasted per household, which has implications for household waste reduction as currently in Ireland 300,000 tonnes of food is wasted [41] and in the UK seven million tonnes of food is wasted [42] annually from households.

TLs had greater cooking confidence and FS confidence than ALs, they currently used more FS, were more open to new food, had a higher Food Safety knowledge, and consumed less fried food, biscuits, chocolate or savoury foods. These results show the positive outcomes learning cooking skills at an early age has on current skills, practices, attitudes and diet quality. Lack of cooking confidence has been shown to act as a barrier to home meal preparation [43] and cooking from scratch [21]. An increased frequency of home meal preparation has been associated with a higher diet quality [44]. Thus, learning CS at a younger age increases cooking confidence, eliminating an identified barrier to cooking and facilitates better diet quality.

This study also reinforced previous findings that the Mother is the most common source of information for CS [27, 45]. In addition this research showed the positive associations ‘Mother only’ as a source of learning had with current cooking and dietary practises. However, it has been reported that at present there is a culinary deskilling [27] of domestic cooks, and a reduction in the number [32] and level [46] of skills used in the production of a meal. This would imply that as the number of mothers (i.e. domestic cooks) cooking at home diminishes, the ability and capacity of current mothers to transfer the necessary skills to the next generation will be limited. Therefore, a reliance on the mother as the main source of CS transmission to the next generation could be detrimental to the learning of CS. Other reported sources, including the educational system, must be considered as potential mechanisms for reskilling current cooks and up-skilling future cooks. Further the importance of family and social influences reported as significant sources of learning, must be incorporated into cooking interventions. A possible method for this would be to include family members as part of the interventions or conducting interventions in a group setting with friends, or interventions reinforced with contact with health care professionals at key points, as is done in the US WIC programme for pregnant women [47].

The above results suggests that the beginning of learning the basics of CS should be in the home environment and/or primary school and be strongly supported in secondary school. These findings are closely aligned with skill formation research which shows the maximum economic and sustainable benefit for investment is through early intervention that must be followed by continued high quality intervention [48]. This study supports and emphasises the recommendation for high-quality, practical and compulsory cooking education for all. This is in line with what has previously been proposed in both the media and academic research, and most recently has been recommended by the World Health Organisation to combat childhood obesity [22, 49–51].

The positive associations of learning CS as a child or teenager was shown on various measures with the exception of fibre intake. AL had a higher intake of fibre than CL or TL, which may have been influenced by the increased awareness of the importance of fibre in the diet of adults [52]. However, as the reported intake of fibre was still below the recommended level, an increased effort on the benefits of dietary fibre should be addressed in all CS interventions regardless of age.

From the above it is clear that learning of CS at an early age has implications on many outcome measures and research areas including health, diet quality, cooking behaviours and self-efficacy, as well as for the educational system.

### Study strengths and limitations

The strengths of this study include diet quality and cooking and food skills data collection from a quota-controlled nationally representative sample of adults living in the UK (NI) and ROI. The overall sample closely matched that of recent census estimations for both NI and ROI (Northern Ireland Census of Population 2011; Republic of Ireland Census of Population 2011). This research used previously validated measures where possible, which improves its comparability and repeatability. The self-reported cooking and food skills abilities assessment tool underwent rigorous development and psychometric testing. All measures developed by the researchers were also tested and had acceptable internal reliability.

Data were self-reported, and therefore may have suffered from memory, response, and social desirability bias. However, where possible this was addressed, for example, in the cooking and food skills ability scales participants only rated their confidence levels if they had reported using a skill, preventing over-inflated confidence scores and reduced response bias. A limitation of this study is that this is a cross-sectional survey, which does not allow for causal interpretations of the data. The subjective nature of the measurement of when the majority of learning occurred may be considered a further limitation, however, as age has not been considered in this area before it is a difficult concept to investigate and for participants to answer. Longitudinal studies following participants who have learned their cooking skills at different stages and measuring their dietary behaviours at a certain age would be needed to reduce the bias. Finally as this study was part of a larger project, potential confounding variables such as living situation, resources and accessibility to learning sources when the participants were growing up were not assessed and should be included in future research to corroborate the current findings.

Nonetheless, as this is the first study exploring the effects of age of learning cooking skills, the results provide a baseline and highlight the need for further empirical longitudinal research into the learning of cooking skills at different ages and learning from different sources.

## Conclusions

Learning CS as a child or a teenager was shown to be positively related to current use of cooking and food skills, cooking practices, cooking attitude and diet quality. This research illustrates that learning CS early in life has potential associations with health, cooking behaviours and food sustainability. In addition the mother was the most commonly named source for past learning and learning from the mother only was linked with greater level of cooking and better dietary practises. Due to the reduction in the number of home cooks this knowledge transfer may not be possible in the future and therefore high quality practical cooking education starting at a younger age is recommended.

## Abbreviations

AL: Adult learners
CL: Child learners
CS: Cooking skills
FS: Food skills
IOI: Island of Ireland
NI: Northern Ireland
ROI: Republic of Ireland
TL: Teen learners
